# Optimal multi-source forecasting of seasonal influenza

**DOI:** 10.1371/journal.pcbi.1006236

**Published:** 2018-09-04

**Authors:** Zeynep Ertem, Dorrie Raymond, Lauren Ancel Meyers

**Affiliations:** 1 Department of Statistics and Data Science, The University of Texas at Austin, Austin, Texas, United States of America; 2 athenaResearch, Watertown, Massachusetts, United States of America; 3 Departments of Integrative Biology and Statistics and Data Science, The University of Texas at Austin, Austin, Texas, United States of America; 4 The Santa Fe Institute, Santa Fe, New Mexico, United States of America; University of New South Wales, AUSTRALIA

## Abstract

Forecasting the emergence and spread of influenza viruses is an important public health challenge. Timely and accurate estimates of influenza prevalence, particularly of severe cases requiring hospitalization, can improve control measures to reduce transmission and mortality. Here, we extend a previously published machine learning method for influenza forecasting to integrate multiple diverse data sources, including traditional surveillance data, electronic health records, internet search traffic, and social media activity. Our hierarchical framework uses multi-linear regression to combine forecasts from multiple data sources and greedy optimization with forward selection to sequentially choose the most predictive combinations of data sources. We show that the systematic integration of complementary data sources can substantially improve forecast accuracy over single data sources. When forecasting the Center for Disease Control and Prevention (CDC) influenza-like-illness reports (ILINet) from week 48 through week 20, the optimal combination of predictors includes public health surveillance data and commercially available electronic medical records, but neither search engine nor social media data.

## Author summary

In the United States, seasonal influenza causes thousands of deaths and hundreds of thousands of hospitalizations. The annual timing and burden of the flu season vary considerably with the severity of the circulating viruses. Epidemic forecasting can inform early and effective countermeasures to limit the human toll of severe seasonal and pandemic influenza. With a growing toolkit of sophisticated statistical methods and the recent explosion of influenza-related data, we can now systematically match models to data to achieve timely and accurate warning as flu epidemics emerge, peak and subside. Here, we introduce a framework for identifying optimal combinations of data sources, and show that public health surveillance data and electronic health records collectively forecast seasonal influenza better than any single data source alone and better than influenza-related search engine and social media data.

## Introduction

Seasonal influenza epidemics annually result in significant global morbidity and mortality [1], and influenza pandemics can cause catastrophic levels of death, social disruption, and economic loss [2]. Early detection and forecasting of both emergence and peak epidemic activity can inform an effective allocation of resources, surge planning, and public health messaging [1, 3–5]. Thus, public health and scientific communities have prioritized the development of influenza forecasting technologies [6–11].

There are a growing number and variety of readily available disease-related data sources that may ultimately be integrated into or even replace traditional systems. The Center for Disease Control and Prevention (CDC) relies on data from two primary national influenza surveillance systems: (1) the U.S. World Health Organization (WHO) and National Respiratory and Enteric Virus Surveillance System (NREVSS) collaborating laboratories (henceforth, WHO US) and (2) the US Outpatient Influenza-like Illness Surveillance Network (ILINet). Recently, Meaningful Use [12], a CDC led effort, is advancing the expansion of syndromic surveillance systems such as ESSENCE to address a broader set of infectious disease surveillance objectives [13–15].

Novel data sources for outbreak surveillance are also arising outside of public health. Notably, researchers at Google launched the Google Flu Trends service (GFT) in 2008 to provide real-time estimates of influenza prevalence based on disease-related search activity [16]. They showed that time series tracking the volumes of influenza-related Google searches closely mirrored influenza data from ILINet. However, it failed to capture the emergence of the 2009 H1N1 pandemic and fell short in subsequent influenza seasons [17–21], resulting in the termination of the program in August 2015 by the company. Epidemic-related data have also been extracted from not only search engines [22] but also interactive web-based applications (e.g., Flu Near You, InfluenzaNet) [23] and online social platforms such as Twitter (e.g., MappyHealth) [24, 25], Facebook [24–29], and Wikipedia [30]. While most of these data sources contain broad information, epidemic related data is *passively* mined and filtered. There are, however, a few participatory systems that *directly* solicit health data from voluntary participants [23]. For example, InfluenzaNet, has over 50 000 volunteers from ten European countries [23]. While many of these sources have been shown, individually, to estimate and predict influenza activity, we have yet to build forecasting models based on systematic comparisons and integration of complementary data.

Given the real-time availability of GFT at multiple geographic scales (from city to continental), many of the early forecasting methods used GFT as a test bed. Notably, Shaman et al. [8] pioneers the use of Kalman filters to predict seasonal GFT dynamics from historical GFT and humidity data and Nsoesie et al. [31] couples a simulation optimization method with a network-based epidemiological model to forecast regional influenza peaks. Another study forecasts GFT from a combination of GFT, temperature, and humidity data in a specific metropolitan area (Baltimore), and demonstrates that the integration of multiple data sources can improve forecast accuracy [7].

More recent forecasting efforts have directly targeted CDC ILINet, rather than GFT, using a variety of predictor data sources. Brooks et al. [6] apply a novel simulation-based Bayesian forecasting framework to forecast one season of ILINet from prior ILINet data. Their method first constructs prior distributions of seasonal flu curves by stochastically combining and transforming features of past flu seasons. As a season emerges, it updates the posterior distribution based on real-time observations and uses importance sampling to generate forecasts. Two other studies forecast ILINet from alternative data sources—one evaluates the predictive performance of Google, Twitter, and Wikipedia, individually [32], and the other considers a multi-linear combination of internet source, digital surveillance, and electronic medical records data [33].

Such data sources vary considerably in both availability and reliability. Some are available in near-real time, whereas others are lagged by days or weeks; some deeply sample geographic or socioeconomic slices of a population, whereas others provide representative but sparse samples of an entire population. In particular, internet and social media data can be misleading, particularly during newsworthy epidemiological events [34–36], but potentially provide a valuable real-time window into emerging events when combined with validated public health or medical data sources. Optimization allows us to systematically balance such trade-offs and quantify the informational content and complementarity of different categories of data. We argue that, for a given forecasting task, candidate data sources should be evaluated and integrated based on clear performance metrics, which may include, for example, measures of forecast accuracy or precision at one or across multiple time points.

Here, we introduce an optimization method for designing robust multi-source epidemic forecasting systems and apply it forecasting seasonal flu in the US. Our framework is intended to be *plug-and-play*, allowing researchers to evaluate large combinations of data sources with respect to their own forecasting model and performance metrics. In our case study, the candidate data sources include thousands of time series data sources from public health surveillance systems, electronic health records systems (EHR), search engines, and other website and social media applications. Our forecasting model is an extension of the flexible Bayesian machine learning method introduced in [6], modified to combine multiple predictors. Finally, our objective function considers overall similarity between historical data and out-of-sample forecasts, averaging across 16 recent flu seasons. Unlike recent multi-source forecasting studies (such as [33]), we present a framework to rigorously evaluate much larger sets of candidate data sources both at the national and regional level and select complementary combinations that maximize forecast performance metrics. This approach not only yields more accurate forecasts, but provides quantitative insight into the relative utility of data sources.

## Materials and methods

### Data sources

#### Forecasting target data

Our forecasting target is the aggregate flu data from ILINet, the CDC national sentinel surveillance system [37]. ILINet tracks weekly counts of patients seeking care for influenza-like-illness, as reported by a sample of health-care providers throughout the US. We obtained national reports between 10/03/1997 and 05/16/2014 from CDC FluView website [12]. We report results on forecasting national-scale ILINet in our main text, and report several state-level forecasts for comparison in Table in S1 Table.

#### Predictor data sources

We consider multiple public health, clinical, and internet data sources as candidate predictors for forecasting seasonal flu, including the CDC ILINet data described above. Below is a brief description of the other data sources included in our study.

*Lab-confirmed influenza cases (WHO US)*: This data includes the percentage of positive tested laboratory analysis of all respiratory specimens reported to CDC from over 400 clinical laboratory facilities located throughout the US and its territories. We use the national-level percentages of all respiratory specimens that test positive for influenza. We obtained this data for the time interval between 05/23/2003 and 05/16/2014 through the FluNet website [38].

*Athena Health flu-related electronic health records data (Athena)*: athenahealth, a for-profit company providing cloud-based services for healthcare providers, supplied weekly data on flu-related patient visits throughout the US from 05/27/11 to 5/16/14. Specifically, we obtained separate time series for six quantities: the numbers of patient visits that included (1) a flu vaccination, (2) flu diagnosis, (3) ILI diagnosis, (4) a flu test (regardless of result), (5) a positive flu test and (6) a flu-related prescription. The data were aggregated at the state-, Health and Human Services (HHS) region-, and national-levels, totalling 435 different influenza-related Athena time series.

*Wikipedia flu-related activity (Wiki Flu)*: This data includes the number of page accesses for the *influenza* page on Wikipedia, a collaboratively written, online, free encyclopedia [30]. Similar to previous studies [36, 39], we collected this data for the time interval between 05/23/2007 and 05/16/2014. Wikipedia receives millions of hits on a weekly basis. We normalized the time series for the influenza page hit values to obtain a standard deviation of one.

*WordPress flu-related blogs (WordPress Flu)*: This data includes the number of new posts related to influenza in each week on WordPress, a free blogging platform with almost 60 million new posts per month [40, 41]. On this platform, users tag posts with keywords to relate them to certain topics. We used a crawling algorithm to count the number of new posts that were tagged with “influenza” for the time interval between 05/23/2003 to 05/16/2014.

*Twitter flu activity (HT US)*: This data includes the percentage of tweets related to influenza infections identified by HealthTweets.org [42] using a simple machine learning classifier [43]. We use their data and categorization to obtain the percentage of influenza-related tweets at the national-level in each week between 05/25/2012 and 05/16/2014.

*HM Athena*: Santillana et al. [33] provide national estimates of the number of patients seeking medical attention for ILI, estimated from athenahealth data. We included this curated data for the time interval between 05/25/2012 and 05/16/2014.

### Hierarchical model selection

**Fig 1:**
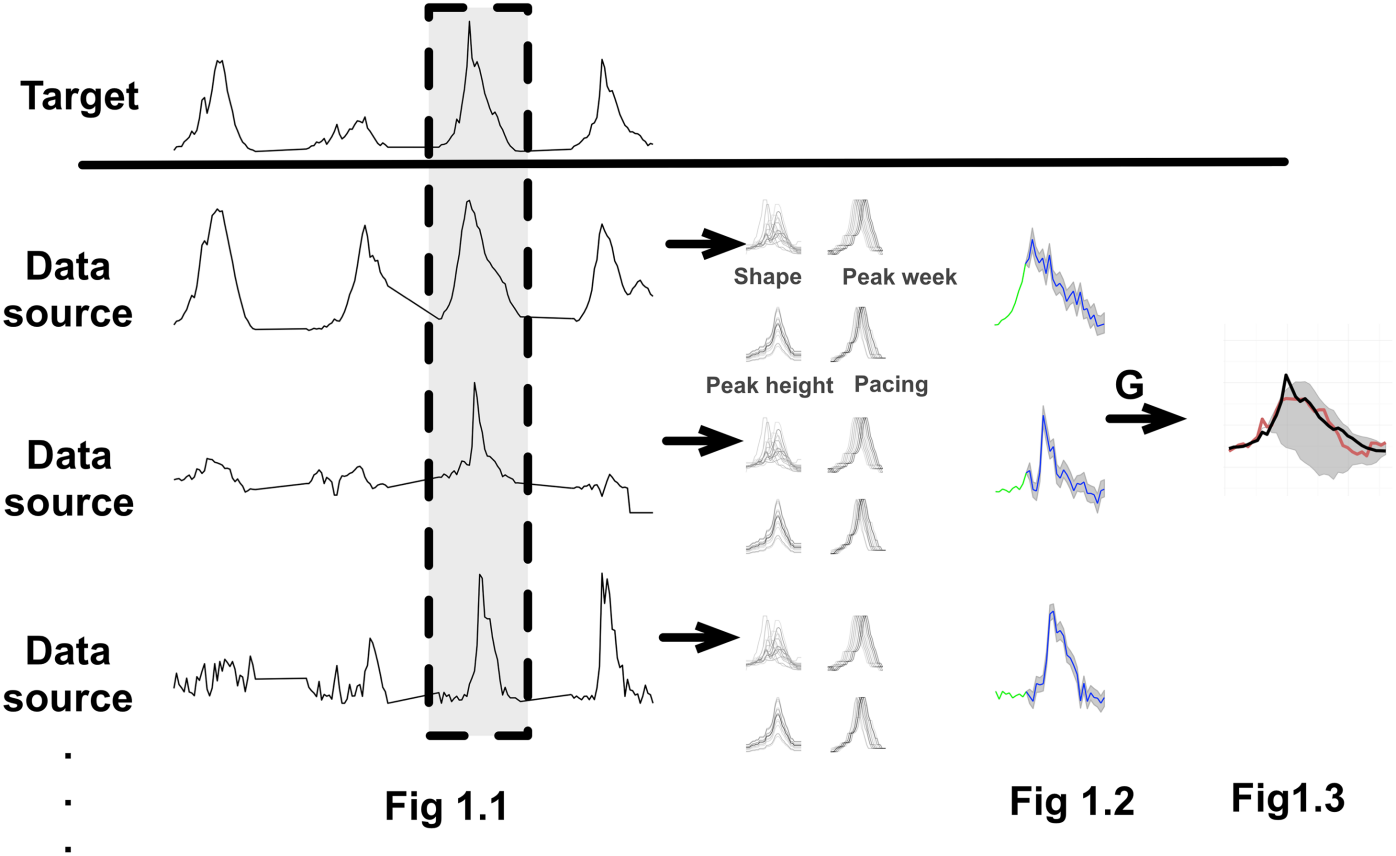
Multi-linear forecast of a historical influenza season. When evaluating a candidate data source, we combine it with previously selected data sources and perform a series of leave-one-out forecasts. Each forecast involves three steps. (1) Align data and remove the *focal* season from all time series (gray band). (2) Make separate Bayes forecasts for each predictor, using the method introduced in [6] (green curves indicate observed weeks and blue curves indicate forecasts). The forecasts are derived from distributions of prototypical curves generated by perturbing and combining characteristics of historic seasons for each candidate data source (shape, pace, peak timing and peak height). (3) Integrate the predictor forecasts into a target forecast (red curve) using the multi-linear model *g* fit to the historical predictor and target data. We evaluate this approach by comparing our target forecasts with the true values of the *focal* season (black curve).

We use greedy optimization with forward selection to iteratively identify combinations of predictor data sources that collectively result in the most accurate forecast for a target data source. Our approach consists of three steps, as shown in Fig 1. First, we individually forecast candidate data sources using an empirical Bayesian framework. Second, we use linear models to combine such individual forecasts into grand forecasts of a target time series. Finally, we build an optimal forecasting system (i.e., collection of predictor data sources) by sequentially adding candidate data sources that most improve the accuracy of historical out-of-sample forecasts of the target. Next sections describe these steps in detail.

#### Forecasting candidate data sources

The target data source is a time series in which distinct epidemics (seasons) can be identified and extracted. In our case, the target is ILINet or one of the other influenza-related time series listed above. Each predictor data source is a historical time series that can be aligned with the target. When forecasting a season, we assume that all predictor data sources can *only* be observed for the first *w* weeks of the season, and then make *n* week long forecasts beginning with the *w + 1*st week of the season. The target data source is assumed to be unobserved for the focal season, unless it is also serving as a predictor.

We use the empirical Bayes framework proposed by Brooks et al. [6] to forecast each predictor time series (from itself). It assumes that future seasons will resemble past seasons with pre-defined transformations. Let **f** denote the seasonal curve for a given data source and season. We assume that the value for the *i*th week is $f\left(i\right)≜f\left(i\right)+\epsilon_{i}$, where *f* is an underlying seasonal trend and *ϵ*_*i*_ ∼ *N*(0, *σ*) is a Gaussian error term.

We build prior distributions for future seasons by combining five key characteristics of observed seasonal time series:

1. **Shape**—the baseline seasonal curve (i.e., *f*)
2. **Peak height**—the maximum value of a seasonal curve (i.e., max(**f**))
3. **Peak week**—the week number during which the peak occurs (i.e., arg max (**f**))
4. **Pace**—the duration that a seasonal curve remains above the baseline before and after its peak
5. **Noise**—the standard deviation of the Gaussian error distribution (i.e., *σ*).

When forecasting season *s* from data source *d*, we estimate prior distributions for each of these characteristics from historical *observed* seasonal data, using all seasons of *d* except *s*: a set of possible shape curves, a range of peak height values, a range of peak week values, and a set of noise values. For example, when making an out-of-sample forecast of the 2007-2008 flu season, we build our prior distributions using all seasons for which data are available preceding and following 2007-2008. For ILINet, this would include all seasons between 2003 and 2014, except 2007-2008; for Athena, this would include only seasons between 2011 and 2014.

We generate (i.e., sample) a curve, ${\hat{f}}_{s}$, from the prior distribution, following five steps. First, we randomly select a shape *f*_0_ from the prior distribution of shapes, which consists of all historical curves smoothed by piecewise quadratic trend filtering [44]. Second, we randomly sample a peak height, *θ*, from *U*(Θ_*m*_, Θ_*M*_) where Θ_*m*_ is the minimum observed peak height and Θ_*M*_ is the maximum observed peak height, and adjust the peak height of the sample curve using $f_{1}\left(i\right)=b+\frac{\theta -b}{\theta_{0}-b}\left(f_{0}\left(i\right)-b\right)$ where *θ*_0_ is the height of *f*_0_ and *b* is the baseline level for data source *d* (e.g., 2% for ILINet). Third, we randomly sample an integer valued peak week *υ* from *U*(ϒ_*m*_, ϒ_*M*_) where ϒ_*m*_ is the earliest observed peak week and ϒ_*M*_ is the latest observed peak week, and shift the values in the sample curve by *f*_2_(*i*) = *f*_1_(*i* − *υ* + *υ*_1_) where *υ*_1_ is the peak week of *f*_1_. Fourth, we sample a pace *π* from *U*[0.75, 1.25] and make our last adjustment to the sample curve with $\hat{f}\left(i\right)=f_{2}(\frac{i-\upsilon }{\pi }+\upsilon )$, following Brooks et al. [6]. Finally, we sample a standard deviation value, $\hat{\sigma }$, for the error distribution, $\epsilon \left(i\right)∼N\left(0,\hat{\sigma }\right)$ from the set of historical noise terms. The noise from historical season *j* is estimated by comparing the actual curve *ζ*_*j*_ to the smoothed curve *f*_*j*_ (using quadratic piecewise trend filter [44]), as given by $\sigma_{j}≜\sqrt{\underset{i}{a}vg{\left(\zeta_{j}\left(i\right)-f_{j}\left(i\right)\right)}^{2}}$. This five-step sampling process yields a curve from the prior defined by $\left(\hat{f},\hat{\sigma }\right)$.

Given observed values for the first *w* weeks of the season, the posterior distribution for a season is determined via importance sampling. We sample *K* values from the prior in the form of $\left({\hat{f}}_{k},{\hat{\sigma }}_{k}\right)$ and calculate the importance, *I*_*k*_, of each by:

$$\begin{array}{c} I_{k}=\prod_{i=1}^{w}P\left(l\left(i\right)|N\left({\hat{f}}_{k}\left(i\right),{\hat{\sigma }}_{k}\right)\right) \end{array}$$ (1)

where *l*(*i*) is the observation at week *i* of the focal season. We calculate the posterior expected values for the remaining seasonal time series, as given by

$$\begin{array}{c} \phi =E\left[f_{s}|l\left(1\right),l\left(2\right),...,l\left(w\right),\left\{{\hat{f}}_{1},{\hat{f}}_{2},...,{\hat{f}}_{K}\right\}\right]=\frac{\sum_{k=1}^{K}I_{k}\times {\hat{f}}_{k}}{\sum_{k=1}^{K}I_{k}}. \end{array}$$ (2)

To obtain credible intervals, we assume that error is distributed normally around the expected values, with posterior standard deviation for week *i* given by

$$\begin{array}{c} \rho \left(i\right)=\sqrt{\frac{\sum_{k=1}^{K}I_{k}\cdot {\left({\hat{f}}_{k}\left(i\right)-\phi \left(i\right)\right)}^{2}}{\sum_{k=1}^{K}I_{k}}}. \end{array}$$ (3)

For each candidate data source, we separately forecast each season *s* and form the prior distribution for *s* using data from all available seasons before and after (but not including) *s*. The uncertainty and quality of forecasts depend on the prior sample size *K*. We used *K* = 100, 000 for optimizing the forecasting systems described herein.

#### Forecasting a target time series

After obtaining forecasts for predictor data sources using the above Bayesian empirical method, we combine them via a linear model to predict the target data source, as given by

$$\begin{array}{c} F\left(s,t\right)≜\beta_{1}\cdot \hat{D_{1}}\left(s,t\right)+\beta_{2}\cdot \hat{D_{2}}\left(s,t\right)+...+\beta_{n}\cdot \hat{D_{n}}\left(s,t\right) \end{array}$$ (4)

where *F*(*s*, *t*) is the value of the target data source in season *s* and time *t* and $\hat{D_{i}}$ refers to the expected forecasted values of predictor *i*. The *β* coefficients are obtained by fitting a regression model to complete historical time series for the target and predictor variables.

To obtain the credible intervals for the target data source, we generate an additional *N* = 10, 000 separate target forecasts, each based on an independent sample from the posterior curves of each of the candidate predictors. The 95% credible interval is then constructed using the 2.5th and 97.5th percentile value at each week of the forecast.

#### Data source selection

We start by choosing the candidate data source that provides the best forecasts on its own. During subsequent rounds of selection, we evaluate each remaining candidate data source by (a) combining it with the previously selected data sources, (b) fitting a new linear model to the combined set of data sources, (c) for each season, calculate individual Bayesian empirical forecasts for each of the predictors, (d) for each season, derive a target forecast from the predictor forecasts using the fit linear model, (e) calculate the average RMSE (as defined in Eq 5) of the resulting forecasts across all seasons. We then select the data source that, when combined with the previously selected data sources, produces the minimum average RMSE. In this way, we can sequentially build a set of complementary predictors that collectively predict the target time series. (See Algorithm in S1 Algorithm for our data selection procedure).

The RMSE objective function favors forecasts that resemble the target ILINet data throughout entire seasons. We initially evaluated other objective functions, including minimization of (1) peak week error, (2) peak magnitude error, (3) both peak week and peak magnitude errors, and (4) RMSE values in a sliding window around the peak week. We found that overall RMSE minimization achieves not only the best season-long accuracy, but comparable predictions of peak timing and magnitude as the more targeted objective functions.

### Evaluating forecasts

We use RMSE to evaluate forecasts and thereby select informative combinations of data sources. It measures the difference between predicted and actual time series, as given by

$$\begin{array}{c} {\text{RMSE}}_{s}\;=\sqrt{\frac{1}{n}\sum_{w=1}^{n}{\left(x_{w}-y_{w}\right)}^{2}} \end{array}$$ (5)

where *x*_*w*_ and *y*_*w*_ denote the observed and predicted values of the target data source, respectively, at week *w* of the season, for *w* = {1, 2, …, *n*}. Post selection, we evaluate the quality of the forecasts using two additional metrics that address the timing and magnitude of the epidemic peak. Specifically, the peak week error (PWE) of a given season is the absolute difference between predicted and actual peak week, as given by

$$\begin{array}{c} {\text{PWE}}_{s}\;=\left|p-\tilde{p}\right| \end{array}$$ (6)

where *p* and $\tilde{p}$ denote the weeks during which the observed and predicted time series, respectively, hit their maximum values. The peak magnitude error (PME) of a given season is the ratio of the absolute difference between the maximum observed and predicted values of the time series and the maximum observed value, as given by

$$\begin{array}{c} {\text{PME}}_{s}=\frac{\left|h-\tilde{h}\right|}{h} \end{array}$$ (7)

where *h* and $\tilde{h}$ denote the maximum values reached by the observed and predicted target time series, respectively.

#### Computing resources

We performed these analyses using Python and R programming languages on a *Macintosh HD* computer with seven 3.1 GHz Intel Core processors and 16 GB RAM. We also used the *Stampede* supercomputer cluster in Texas Advanced Computing Center (TACC) to parallelize the computation of Bayesian forecasts of candidate data sources.

## Results

We analyzed several different sets of candidate data sources, with the goal of identifying subsets of data sources that provide accurate and timely forecasts of ILINet. For each round of data evaluation, we separately predicted each season between 1997 and 2014, excluding the 2009-2010 H1N1 pandemic. For simplicity, we assumed that all 16 seasons span from the 40th calendar week of a given year to the 20th calendar week of the subsequent year. For each season in each data source, we assume that we observe values during the first nine weeks of the season (i.e., the 40th through 48th calendar week) and then forecast ILINet levels for the remainder of the flu season.

Each experiment resulted in an optimized surveillance system, that is, a list of data sources prioritized by the order in which they were selected during optimization. We compare the optimized surveillance systems using three metrics that evaluate the accuracy of the overall (RMSE) and peak (PWE and PME) forecasts.

First, we consider an optimized system consisting of five data sources selected from among all 453 local, regional and national data sources, and compare it to two baseline systems–one using only ILINet to forecast itself and another using a combination of ILINet and WHO laboratory data to forecast ILINet (Table 1). ILINet is selected as the single most informative predictor when evaluated in conjunction with only WHO laboratory data or with all 453 available sources. The fully optimized system combines ILINet with WHO and three Athena state- and regional-level data sources (no internet-based data sources is chosen), suggesting that proprietary electronic medical record data may provide a more reliable source of real-time epidemiological data than freely available internet source data. In comparing the ILINet plus WHO system to the fully optimized system (All), we find that Athena data improves performance only marginally relative to the addition of all four data sources, which together reduce the historical RMSE by roughly 15%.

**Table 1:** Performance of baseline and optimized surveillance systems.

Candidate sources	Selected sources	RMSE (%ILI)		PWE (weeks)		PME (%ILI)	
		Mean	[Min, Max]	Mean	[Min, Max]	Mean	[Min, Max]
ILINet	ILINet US	0.66	[0.26,1.10]	2.43	[0, 6]	0.24	[0.008,0.71]
ILINet & WHO	ILINet US WHO US	0.63	[0.26,0.98]	2.31	[0, 6]	0.24	[0.009,0.66]
All	ILINet US WHO US Athena FluResultVisit IL Athena FluResultVisit GA Athena PositiveResult% HHS 08	0.56	[0.21,0.96]	1.75	[0, 6]	0.19	[0.04, 0.39]
All national (US)	ILINet US WHO US Athena ILIVisit US Athena ILI% US WordPress Flu	0.60	[0.26,0.98]	2.12	[0, 6]	0.21	[0.02, 0.40]
All national without ILINet	WHO US Athena FluResultVisit US Athena FluRXVisit US Athena FluVisit US Athena ILIVisit US	0.64	[0.18, 1.30]	2.37	[0, 8]	0.21	[0.002,0.45]
All national without ILINet & WHO	Athena ILI% US Wiki Flu HM Athena WordPress Flu Athena FluResultVisit US	0.87	[0.29,1.75]	10.81	[0, 19]	0.44	[0.15,0.71]

Data sources were selected based on accuracy (RMSE) of 16 out-of-sample retrospective flu season forecasts (1997-2014, excluding the 2009-2010 H1N1 pandemic), and listed in order of selection. **All** and **National (US)** includes 453 and 13 candidate data sources, respectively. Mean, minimum, and maximum values are calculated over the 16 seasons.

The optimization selected Athena data from HHS region 8, Illinois, and Georgia, from among all 435 Athena candidate time series. To assess the value of such local, state and regional data, we conducted an additional experiment, restricting the selection to only US-level candidate data sources. The resulting system includes two national Athena data sources (i.e., absolute and percent ILI visits across all facilities) and WordPress flu activity (Table 1). It yields better forecasts than the public health baselines, but is inferior to the optimized system that includes state and regional data.

While ILINet and WHO data are consistently selected as the most informative data sources, they tend to have greater time lags than some of the other *real-time* candidate data sources. To evaluate the viability of a real-time system using alternative national-level data, we optimized two additional systems, one excluding ILINet and the other excluding both ILINet and WHO data. Without ILINet, WHO is selected as the single most informative source and combined with four different national-level Athena data sources tracking flu-related visits and prescriptions (Table 1). The forecasts decline only slightly relative to systems that include ILINet. However, when both ILINet and WHO data are excluded, the expected performance drops considerably. For comparison, we optimized systems for forecasting state-level ILINet (California, New York, and Texas), and found that national-level surveillance data (ILINet and WHO US) are always selected among the top three most informative data sources, with forecasts enhanced by a variety of state and regional athenahealth variables. (See Table in S1 Table)

### Forecasting accuracy

**Fig 2:**
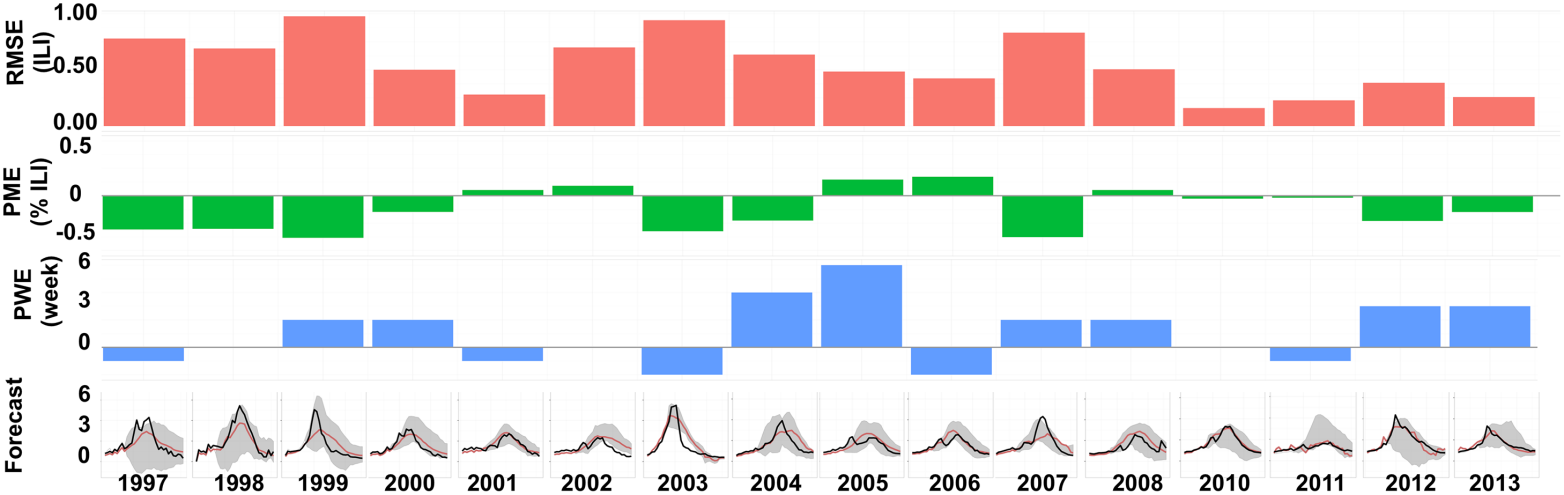
Forecasts of historical flu seasons from 1997-1998 through 2013-2014 (excluding 2009-2010) by the optimized five-source surveillance system. The system includes ILINet, WHO, and three Athena data sources. Forecast performance is summarized in top rows of graphs, by RMSE (red), PWE (green), and PME (blue). The bottom row compares the forecasted (red) and actual (black) times series with 95% credible intervals (gray). Vertical dashed lines indicate the last week of the observational periods, after which all predictor and target data are forecasted.

The best five-source system (optimized from all available data sources) consistently produces accurate historical out-of-sample forecasts, as shown in Fig 2. After observing only the first nine weeks of the flu season, the system is able to predict the remaining 24 weeks of the season with an average RMSE under 1%. The forecasted 95% credible interval contained the historical ILINet value in 87% of all weeks across all 16 forecasts. However, the 2002-2003 and 2003-2004 forecasts capture the peaks but considerably overestimate prevalence towards the ends of the seasons (12 weeks out of 24 lie outside the 95% credible interval). Excluding these two seasons, 92.9% of all historical weeks fall within the forecasted 95% interval. In the system optimized from all national-level data sources except ILINet, accuracy drops to 66% of all historical weeks contained in the credible intervals. (See S1 Fig for detailed results).

Although these systems were optimized solely to minimize RMSE, the resulting forecasts perform quite well with respect to predicting the timing and magnitude of the epidemic peak. In over 85% of the seasons, the forecasts predict the peak to occur within two weeks of the actual peak; in over 85%, the predicted height of the peak is within 20% of its actual height. Since the Athena predictors are only available between 2011 and 2014, they provide no information for the first 13 of the 16 seasons. Consequently, we see a reduction in RMSE for the three most recent forecasts.

**Fig 3:**
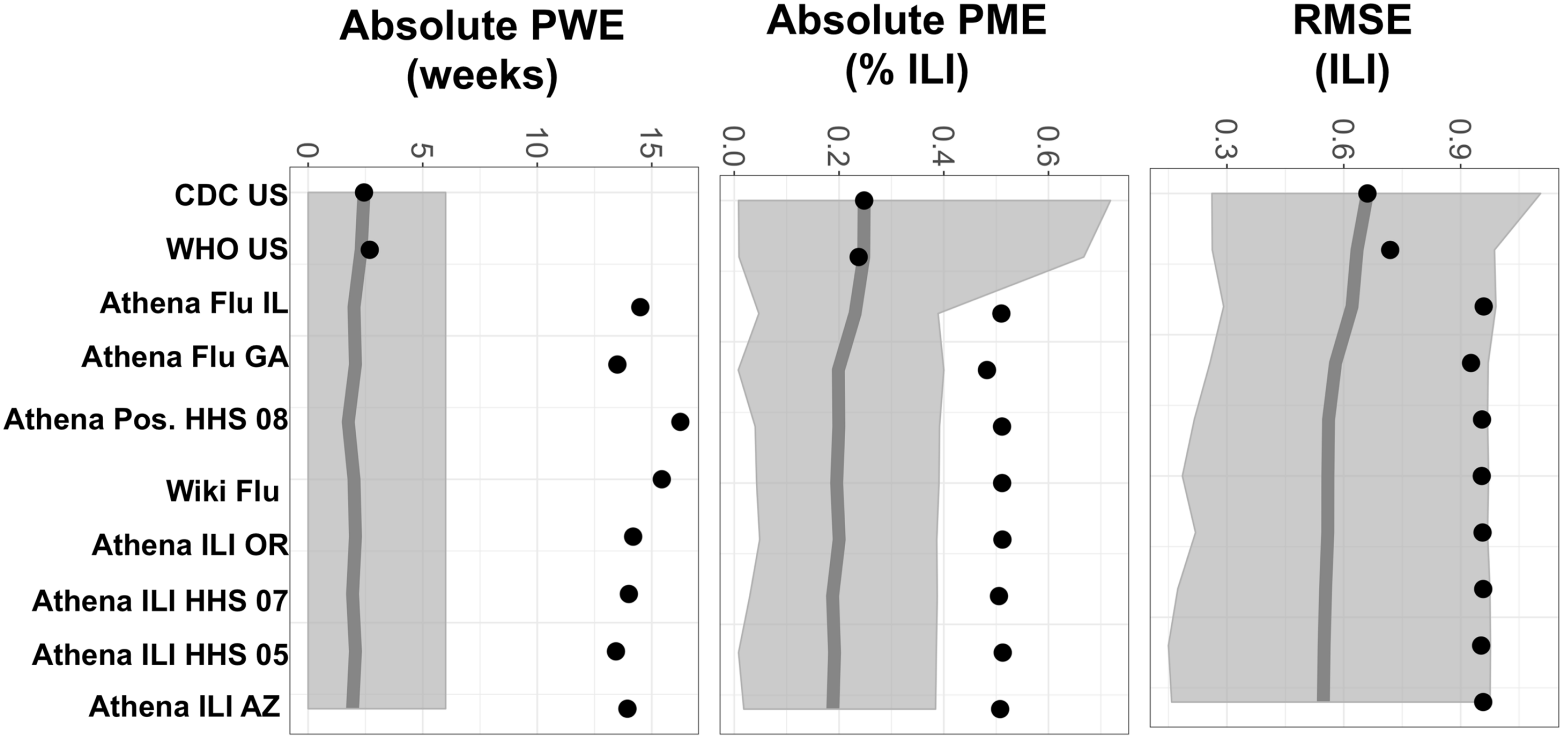
Performance curves for the first ten selected data sources. The system was built through the sequential addition of data sources to minimize RMSE, as listed from left to right along the x-axis. Graphs show the changing performance of the growing system, where points indicate the quality (mean RMSE, PWE, or PME) of forecasts made using all data sources to the left of and including the given x-axis label. Circles indicate individual performance of selected data sources; shading indicates performance range across the 16 seasons tested.

Performance curves for this optimized system indicate that additional data sources, beyond the five included, are not expected to improve performance considerably, according to our empirical results show in Fig 3. On their own, ILINet and WHO are the strongest predictors of future ILINet activity. Although the Athena data sources exhibit poor individual performance, they substantially improve forecast accuracy when combined with ILINet and WHO. The hierarchical selection method was thus able to integrate *complementary* data sources into a multi-source system that is expected to provide more reliable forecasts than single-source systems. This is also true for systems which exclude ILINet and WHO as candidate predictors. (See S2 Fig for detailed results).

We also build out-of-sample forecasts of ILINet using ILINet and WHO as predictors, using only (1) three years (2011-2014) and (2) five years of training data (2008-2014) to build the Bayesian prior distributions. In the original out-of-sample forecasts, we used 15 of the 16 available seasons to build priors for forecasting the remaining season. (See S3 and S4 Figs for more details). Performance increased with the duration of the training data, with average RMSE decreasing from 0.69 to 0.64 to 0.56 as we increase the training period from three to five to fifteen years. However, even the poorest set of forecasts (based on three years of training) are decent. In addition, we note that the original experiments selected Athena Health data as highly informative predictors, despite only being available for three years (2011-2014).

## Discussion

There are a growing number of powerful methods for forecasting seasonal and pandemic flu (e.g. [6, 45]). To achieve earlier and more accurate predictions of epidemic emergence, growth, peaks and burden, researchers are developing sophisticated statistical methods–some adapted from mature forecasting sciences like meteorology [8]–and creatively leveraging diverse sources of predictor data. The increasing public availability of disease-related data sources is promising yet daunting, with annually, hundreds of thousands of influenza-related tweets [42], several millions of page hits on Wikipedia to influenza-related pages [30], thousands of influenza-related blog posts on Wordpress [40] and hundreds of thousands of hospital and clinic visits. While many studies have demonstrated the promise of surveillance [46] and forecasting from novel data sources [33], we do not yet have rigorous methods for evaluating the utility of such data or identifying effective combinations of data for particular models and forecasting goals.

Over several years, we have developed a general framework for addressing exactly this challenge [20, 46, 47]. For any public health surveillance goal, the approach is designed to systematically evaluate up to thousands of candidate data sources and identify complementary combinations of predictors that achieve the stated goal. For example, we have identified optimal zip codes for seasonal flu surveillance and early detection of pandemic flu in Texas [48], selected informative clinics for dengue surveillance in Puerto Rico [47], and developed software for optimal selection and integration of surveillance data sources for the Defense Threat Reduction Agency’s (DTRA’s) Biosurveillance Ecosystem (BSVE) [49].

In this study, we have used this framework to design multi-source surveillance systems for accurate forecasting of seasonal influenza, and, in the process, rigorously assess the performance and complementarity of diverse data sources. To do so, we combined two previously published methods. The first is an empirical Bayes strategy for forecasting seasonal flu from a single data source [6]. Rather than imposing strong assumptions about transmission dynamics, it assumes that the forecasting target (typically, the currently emerging flu season) will roughly resemble past seasons in terms of the shape, peak week, peak magnitude, and pace of the epidemic curve. By combining and perturbing these features from prior seasonal data, we simulate distributions of plausible (hybrid) flu curves. Then, as a season unfolds, we predict future weeks by extrapolating from variates that most resemble recent activity. To forecast flu (target) from *multiple* data sources (predictors), we make empirical Bayes forecasts of each predictor separately and combine them into a target forecast using a linear model previously fit to historical predictor and target data. The second method is a greedy optimization that sequentially selects a maximally informative set of data sources to achieve a specified goal [47, 50]. In our case, the candidate providers are a diverse set of public health, commercial health-care, internet query and social media data sources. Our public health goal is accurate forecasting of seasonal flu starting in calendar week 48.

The field has primarily focused on the development of statistical models that predict seasonal dynamics on multiple geopolitical scales, and only secondarily considered the quality of predictor data. Test bed data are often selected based on convenience. Until recently, Google Flu Trends data was free and abundant at multiple scales, and thus a popular choice [7, 10, 20, 31]. A few studies have integrated multiple different types of data and shown that, for short-term forecasting (one to three weeks ahead), the combination of all independent flu predictors performs better than using single source [33]. However, they have not systematically optimized the combination of data sources or quantified their relative contributions to forecast accuracy, as we have done here. Our study confirms that multi-source forecasting can outperform single-source forecasting, but only when complementary sources are identified and systematically integrated.

We optimized forecasting models from three classes of data–traditional public health surveillance data, electronic health records (EHR) from a data services company, and data aggregated from the influenza-related internet search and social network activity. A priori, each has pros and cons. Official surveillance systems are designed for the purpose of monitoring and predicting flu activity, and thus may provide more accurate and robust signals than the alternatives. However, surveillance data tends to be sparse and time-lagged. Internet source data can be abundant and immediately available, but provides only correlated activity that can be highly susceptible to extrinsic perturbations such as media events and modifications to source websites [34, 35]. EHR data has the combined advantages of real-time availability and access to multi-dimensional flu data at various geographic scales. However, it is not freely available and may require statistical corrections for sampling biases.

Our analyses provide quantitative insights into harnessing these trade-offs for forecasting. First, when data sources are evaluated individually, we find that public health surveillance data yields the most accurate forecasts, followed by EHR data, and internet-source data trailing far behind. Second, optimized combinations of data sources (with or without ILINet) provide far better forecasts than any individual data source alone. Third, EHR data are always selected before internet-source data to augment public health data, suggesting that EHR’s provide a more valuable source of complementary information. Forth, when CDC and WHO data are excluded, the optimal EHR and internet-source systems are unable to achieve comparable forecasting performance. Fifth, state-level EHR data improves forecasts significantly more than national-level EHR data.

While we believe that these insights are robust, they may reflect specific assumptions of our model, and not apply to other diseases, forecasting methods, or objective functions. First, the superior performance of the public health data source is likely biased by our choice of ILINet as the *gold standard* forecasting target. If we had instead sought to forecast athenahealth or GFT time series, these data sources may have been selected as their own top predictors. However, we believe that this choice of target is justified, as it is the only data source specifically designed to estimate flu prevalence in the US. Along with WHO it always selected as a top predictor for selected level forecasts. Second, we follow Brooks et al. [6] in assuming uniform distributions for peak height and peak week, constrained by historical observations. This might limit forecasting accuracy for seasons with atypically high, low, early or late peaks. To address this, one could assume distributions that include low probability extreme departures from past seasons.

We emphasize that this framework is designed to select optimal combinations of data sources for any combination of predictor data sources, multi-linear forecasting method and objective function. As a case study, we built optimal combinations of data sources for forecasting seasonal flu using a published univariate Bayesian empirical framework ([6]) that we extended to forecast with multiple data sources. The optimized systems provide reliable forecasts of the overall seasonal trends and epidemic peak, in most of the 16 historical out-of-sample evaluations. The data-driven selection of informative predictors revealed that public health surveillance data is invaluable for flu forecasting, and that, when rigorously integrated into forecasting models, proprietary electronic health record data can significantly increase accuracy, to a greater degree than freely available internet data. The same optimization framework, forecasting method and RMSE objective function could be readily applied to designing high performing multi-linear forecasting systems for other diseases, for which we have amble historic data, such as Dengue [51–54] and Chikungunya [55]. By modifying the objective function, we can alternatively build systems for forecasting early transmission dynamics or clinical severity of emerging outbreaks.

## Supporting information

S1 Algorithm Hierarchical data source selection. (PDF)

S1 Table Data selected for forecasting ILINet in three US states. (PDF)

S1 Fig Historical flu forecasts from 1997-1998 through 2013-2014 (excluding 2009-2010) from two of the optimized five-source systems. The ‘All’ system was optimized from all candidate data sources; the ‘All national without ILINet’ system was optimized from all national-scale data sources except ILINet. These correspond to the third and fifth systems listed in Table 1, respectively. Plots show the actual (black) and forecasted (red) time series with 95% credible intervals (gray). Across all 16 out-of-sample forecasts, we calculated the proportion of weeks in which the forecasted 95% credible interval contains the historical ILINet value, and found that the ‘All’ and ‘All national without ILINet’ systems achieved 87% and 66% accuracy, respectively.
(PDF)

S2 Fig Performance curves for the first ten selected data sources when all possible data sources are included as candidate predictors (All), when ILINet is excluded (All without ILINet), and when both ILINet and WHO are excluded (All without ILINet and WHO). The system was built through the sequential selection of data sources that minimize average RMSE across 16 out-of-sample forecasts. Selected data are listed in order of inclusion from left to right along the x-axis. Performance is indicated along y-axis in terms of RMSE, with open circles indicating individual performance of selected data sources, and closed circles and shading indicating the mean and range in performance across all 16 out-of-sample forecasts.
(PDF)

S3 Fig Forecasting ILINet from ILINet and WHO predictors, based on a three-year training period (2011-2014). In the original forecasts, we used 15 of the 16 available seasons to build Bayesian priors and then forecasted the remaining season. Here, we use only three seasons to train the model and then forecast the preceding 13 seasons. The average RMSE across these forecasts is 0.69, which is considerably poorer than the average RMSE of 0.56 achieved with the original fifteen-year training periods.
(PDF)

S4 Fig Forecasting ILINet from ILINet and WHO predictors, based on a five-year training period (2008-2014). In the original forecasts, we used 15 of the 16 available seasons to build Bayesian priors and then forecasted the remaining season. Here, we use only five seasons to train the model and then forecast the preceding 11 seasons. These forecasts have an average RMSE of 0.64, compared to average RMSE’s of 0.56 for the original fifteen-year training period and 0.69 for the three-year training period shown in Fig. S3 Fig.
(PDF)
