# Supplementary material for: Optimal multi-source forecasting of seasonal influenza
Source: PLoS Comput Biol. 2018 Sep 4;14(9):e1006236. doi: 10.1371/journal.pcbi.1006236 (PMC6138397; doi:10.1371/journal.pcbi.1006236)
Supplement: S1 Algorithm — (PDF) [file pcbi.1006236.s001.pdf]

1 Pseudo-code for hierarchical selection of data sources for influenza forecasting

S1 Algorithm. Hierarchical data source selection.

1: **Given** : Set of predictor data sources $\mathbf{D}$ and forecasting target G
2: $\boldsymbol{D_{chosen}} : \{D_1, D_2, D_3, \cdots, D_{n-1}\}$
3: $\boldsymbol{D_{remaining}} : \boldsymbol{D} \setminus \boldsymbol{D_{chosen}}$
4: To choose D_n,
5: **for** $q \in \boldsymbol{D_{remaining}}$ **do**
6:     Fit $\Omega_q : G \sim D_1 + D_2 + \cdots + D_{n-1} + q$ on the full historical time series.
7:     $\boldsymbol{S}$: historical seasons of G
8:     **for** $s_i \in \mathbf{S}$ **do**
9:         **for** $d \in \boldsymbol{D_{chosen}} \cup q$ **do**
10:             Make Bayes' forecast of s_i for d from week w, with $(\mathbf{S} \setminus s_i)$ as priors.
11:         Apply Ω_q to forecast G for season s_i from forecasts generated in Step 10.
12:         Score forecast from Step 11 by comparison to actual G (call this score $\sigma(q, i)$).
13:     Calculate the grand score $\sum_{s_i} \sigma(q, i)$.
14: Choose q that maximizes the grand score.
15: Set $D_n = q$.
