# Supplementary material for: Optimal multi-source forecasting of seasonal influenza
Source: PLoS Comput Biol. 2018 Sep 4;14(9):e1006236. doi: 10.1371/journal.pcbi.1006236 (PMC6138397; doi:10.1371/journal.pcbi.1006236)
Supplement: S1 Table — (PDF) [file pcbi.1006236.s002.pdf]

# 1 Forecasting state-level influenza time series

**S1 Table. Data selected for forecasting ILINet in three US states.**

| California | New York | Texas |
|---|---|---|
| ILINet US | ILINet US | ILINet US |
| Athena PositiveResult% IN | Athena FluResultVisit CT | Athena FluResultVisit FL |
| WHO US | WHO US | WHO US |
| Athena PositiveResult% OH | Athena ILIVisit NE | Athena PositiveResult% HHS 03 |
| Athena FluResultVisit NJ | Athena FluResultVisit GA | Athena ILIVisit SC |
| Athena FluResultVisit AZ | Athena FluResultVisit CO | Athena ILI% HHS 04 |
| Wiki Flu | Athena ILIVisit OR | Athena FluVisit AL |
| Athena FluVisit LA | Athena FluResultVisit NC | Athena ILIVisit HHS 09 |
| Athena FluResultVisit GA | Athena FluResultVisit AK | Athena ILIVisit MS |
| Athena FluResultVisit AL | Athena IL% NC | Athena FluRXVisit TX |

We conducted separate optimization experiments for forecasting California, New York, and Texas ILINet activity. Each selected from among all 453 candidates data sources. The table lists the top ten in the order of their selection.
