# Supplementary material for: Optimal multi-source forecasting of seasonal influenza
Source: PLoS Comput Biol. 2018 Sep 4;14(9):e1006236. doi: 10.1371/journal.pcbi.1006236 (PMC6138397; doi:10.1371/journal.pcbi.1006236)
Supplement: S1 Fig — The ‘All’ system was optimized from all candidate data sources; the ‘All national without ILINet’ system was optimized from all national-scale data sources except ILINet. These correspond to the third and fifth systems listed in Table 1, respectively. Plots show the actual (black) and forecasted (red) time series with 95% credible intervals (gray). Across all 16 out-of-sample forecasts, we calculated the proportion of weeks in which the forecasted 95% credible interval contains the historical ILINet value, and found that the ‘All’ and ‘All national without ILINet’ systems achieved 87% and 66% accuracy, respectively. (PDF) [file pcbi.1006236.s003.pdf]

1 Forecast comparison

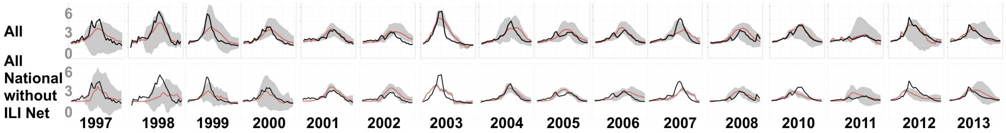

S1 Fig. Historical flu forecasts from 1997-1998 through 2013-2014 (excluding 2009-2010) from two of the optimized five-source systems. The 'All' system was optimized from all candidate data sources; the 'All national without ILINet' system was optimized from all national-scale data sources except ILINet. These correspond to the third and fifth systems listed in Table 1, respectively. Plots show the actual (black) and forecasted (red) time series with 95% credible intervals (gray). Across all 16 out-of-sample forecasts, we calculated the proportion of weeks in which the forecasted 95% credible interval contains the historical ILINet value, and found that the 'All' and 'All national without ILINet' systems achieved 87% and 66% accuracy, respectively.
