# Supplementary material for: Optimal multi-source forecasting of seasonal influenza
Source: PLoS Comput Biol. 2018 Sep 4;14(9):e1006236. doi: 10.1371/journal.pcbi.1006236 (PMC6138397; doi:10.1371/journal.pcbi.1006236)
Supplement: S2 Fig — The system was built through the sequential selection of data sources that minimize average RMSE across 16 out-of-sample forecasts. Selected data are listed in order of inclusion from left to right along the x-axis. Performance is indicated along y-axis in terms of RMSE, with open circles indicating individual performance of selected data sources, and closed circles and shading indicating the mean and range in performance across all 16 out-of-sample forecasts. (PDF) [file pcbi.1006236.s004.pdf]

1 Performance of systems excluding ILINet and WHO as predictors

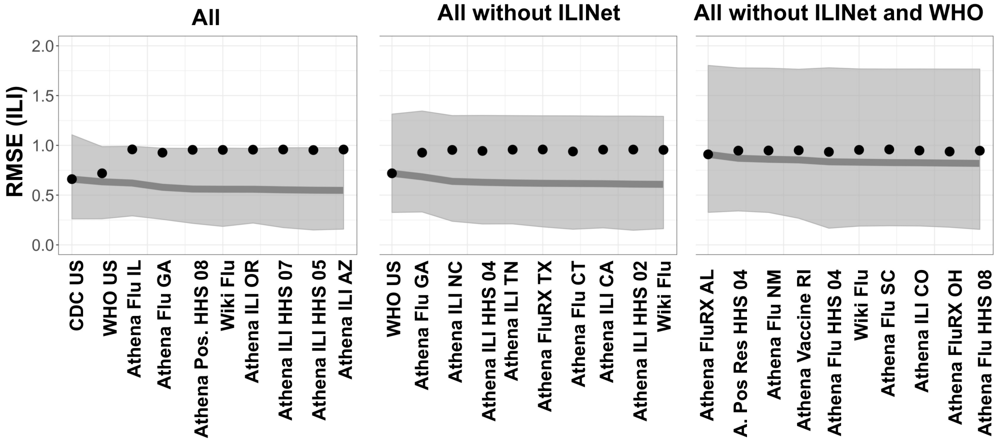

S2 Fig. Performance curves for the first ten selected data sources when all possible data sources are included as candidate predictors (All), when ILINet is excluded (All without ILINet), and when both ILINet and WHO are excluded (All without ILINet and WHO). The system was built through the sequential selection of data sources that minimize average RMSE across 16 out-of-sample forecasts. Selected data are listed in order of inclusion from left to right along the x-axis. Performance is indicated along y-axis in terms of RMSE, with open circles indicating individual performance of selected data sources, and closed circles and shading indicating the mean and range in performance across all 16 out-of-sample forecasts.
