# Supplementary material for: Optimal multi-source forecasting of seasonal influenza
Source: PLoS Comput Biol. 2018 Sep 4;14(9):e1006236. doi: 10.1371/journal.pcbi.1006236 (PMC6138397; doi:10.1371/journal.pcbi.1006236)
Supplement: S3 Fig — In the original forecasts, we used 15 of the 16 available seasons to build Bayesian priors and then forecasted the remaining season. Here, we use only three seasons to train the model and then forecast the preceding 13 seasons. The average RMSE across these forecasts is 0.69, which is considerably poorer than the average RMSE of 0.56 achieved with the original fifteen-year training periods. (PDF) [file pcbi.1006236.s005.pdf]

1 Prior calculated using data from limited seasons

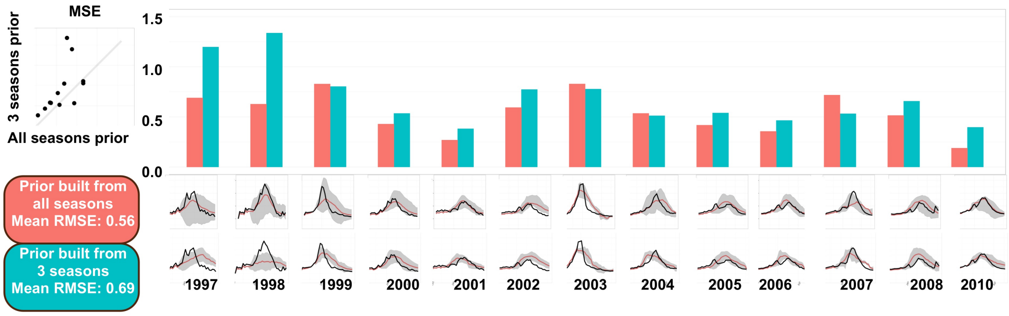

S3 Fig. Forecasting ILINet from ILINet and WHO predictors, based on a three-year training period (2011-2014). In the original forecasts, we used 15 of the 16 available seasons to build Bayesian priors and then forecasted the remaining season. Here, we use only three seasons to train the model and then forecast the preceding 13 seasons. The average RMSE across these forecasts is 0.69, which is considerably poorer than the average RMSE of 0.56 achieved with the original fifteen-year training periods.
