# Supplementary material for: Optimal multi-source forecasting of seasonal influenza
Source: PLoS Comput Biol. 2018 Sep 4;14(9):e1006236. doi: 10.1371/journal.pcbi.1006236 (PMC6138397; doi:10.1371/journal.pcbi.1006236)
Supplement: S4 Fig — In the original forecasts, we used 15 of the 16 available seasons to build Bayesian priors and then forecasted the remaining season. Here, we use only five seasons to train the model and then forecast the preceding 11 seasons. These forecasts have an average RMSE of 0.64, compared to average RMSE’s of 0.56 for the original fifteen-year training period and 0.69 for the three-year training period shown in Fig. S3 Fig. (PDF) [file pcbi.1006236.s006.pdf]

1 Performance of systems excluding ILINet and WHO as predictors

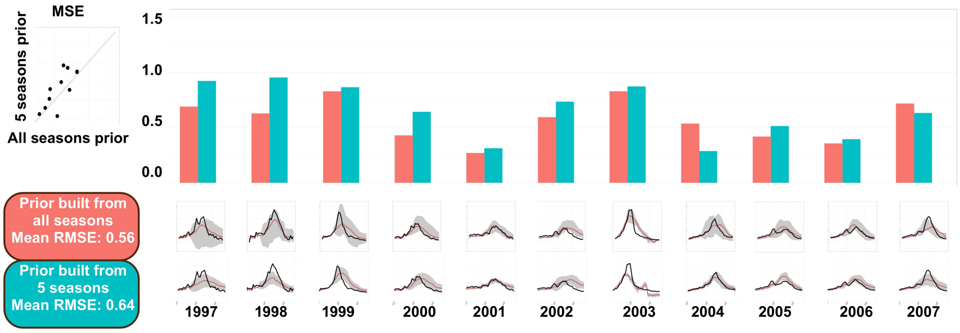

S4 Fig. Forecasting ILINet from ILINet and WHO predictors, based on a five-year training period (2008-2014). In the original forecasts, we used 15 of the 16 available seasons to build Bayesian priors and then forecasted the remaining season. Here, we use only five seasons to train the model and then forecast the preceding 11 seasons. These forecasts have an average RMSE of 0.64, compared to average RMSE's of 0.56 for the original fifteen-year training period and 0.69 for the three-year training period shown in Figure S3 Fig.
